# The Green Tea Catechin Epigallocatechin Gallate Ameliorates Graft-versus-Host Disease

**DOI:** 10.1371/journal.pone.0169630

**Published:** 2017-01-19

**Authors:** Sabine Westphal, Aleixandria McGeary, Sandra Rudloff, Andrea Wilke, Olaf Penack

**Affiliations:** Department of Hematology, Oncology and Tumorimmunology, Charité Campus Virchow, Berlin, Germany; Hospital Universitario de Salamanca, SPAIN

## Abstract

Allogeneic hematopoetic stem cell transplantation (allo-HSCT) is a standard treatment for leukemia and other hematologic malignancies. The major complication of allo-HSCT is graft-versus-host-disease (GVHD), a progressive inflammatory illness characterized by donor immune cells attacking the organs of the recipient. Current GVHD prevention and treatment strategies use immune suppressive drugs and/or anti-T cell reagents these can lead to increased risk of infections and tumor relapse. Recent research demonstrated that epigallocatechin gallate (EGCG), a component found in green tea leaves at a level of 25–35% at dry weight, may be useful in the inhibition of GVHD due to its immune modulatory, anti-oxidative and anti-angiogenic capacities. In murine allo-HSCT recipients treated with EGCG, we found significantly reduced GVHD scores, reduced target organ GVHD and improved survival. EGCG treated allo-HSCT recipients had significantly higher numbers of regulatory T cells in GVHD target organs and in the blood. Furthermore, EGCG treatment resulted in diminished oxidative stress indicated by significant changes of glutathione blood levels as well as glutathione peroxidase in the colon. In summary, our study provides novel evidence demonstrating that EGCG ameliorates lethal GVHD and reduces GVHD-related target organ damage. Possible mechanisms are increased regulatory T cell numbers and reduced oxidative stress.

## Introduction

Allogeneic hematopoietic stem cell transplantation (allo-HSCT) is the only curative treatment option for many patients suffering from leukemia and other hematological malignancies. Unfortunately, many patients die after allo-HSCT due to graft-versus-host disease (GVHD), a progressive inflammatory disease. GVHD is an immunological reaction of donor cells against the recipient, mainly driven by donor T cells. Typical target organs involved in GVHD are the skin, the liver and the gut, where donor T cells induce severe damages of the organs resulting in consecutive loss of organ function.

Standard prophylaxis and therapy of GVHD are immunosuppressive drugs with profound inhibitory effects on allogeneic T cells. The disadvantages of using immunosuppressive drugs after allo-HSCT is an increased risk of potentially life threatening infects and unwanted suppression of anti-tumor effects (graft-versus-tumor, GVT). Therefore, there is a medical need for new therapeutic approaches against GVHD.

Epigallocatechin gallate (EGCG) is a polyphenol which is found in high concentration in the dry leaves of green and white tea and, in smaller quantity, in black tea. [1] We became interested in investigating EGCG during GVHD because of our recent results on successful prevention of tissue damage by ECGC in a murine model of colitis. [2] EGCG has several *in vivo* effects, these include three main mechanisms of how it could potentially reduce GVHD. [3–5] First, GVHD is associated with reduced regulatory T cell numbers in target organs and adoptive therapy of regulatory T cells can be used to prevent GVHD. [6] Several reports indicate that EGCG attenuate inflammatory diseases like arthritis and experimental autoimmune encephalomyelitis (EAE) by its influence on regulatory T cells. [7, 8] Second, GVHD and tumor growth are associated with neovascularization and the inhibition of neovascularization has been used therapeutically to improve patient survival after allo-HSCT. [9, 10] It has been demonstrated that EGCG inhibits neovascularization in different cancers [11] and inflammatory diseases. [12] Third, GVHD is associated with high levels of reactive oxygen species (ROS) [13] and the reduction of ROS is recognized as a therapeutic principle to reduce inflammation. [14] It was shown that EGCG reduces the amount of ROS in inflammatory diseases, such as in an inflammatory model in human corneal epithelial cells. [15] The objective of the present study was to test our hypothesis that EGCG is useful in ameliorating GVHD.

## Methods

### Mice

Female C57BL7/6 mice, female B6D2F1 mine and female and male LP/J mice were purchased from Charles River Laboratories (Sulzfeld,Germany). Animals were 10–12 weeks old and had at least a weight of 23 g. Mice housed in the Charité University Hospital Animal Facility under pathogen-free controlled conditions and 12hr-light/dark cycle. Mice received normal chow and autoclaved hyperchlorinated drinking water. All experiments were approved by the Regional Ethics Committee for Animal Research (State Office of Health and Social Affairs Berlin, Permit number G0414/09). All efforts were made to minimize suffering. For all experiments, mice were monitored daily. Mice were individually scored twice a week for five clinical parameters (posture, activity, fur, skin and weight loss) on a scale from 0–2 (S1 Table). Clinical GVHD score was assessed by summation of these parameters. Animals were sacrificed when exceeding a score of 2 for any one parameter or a score of 6 in total. Mice were euthanized by exposure to carbon dioxide. No animals died without euthanasia before meeting the humane endpoint criteria.

### Conditioning, allogeneic stem cell transplantation (allo-BMT) and treatment with EGCG/ Quercetin

Two different murine models were used. The major mismatch model C57BL/6→B6D2F1 which has a high and early mortality rate and is used to examine survival and the minor mismatch model LP/J→C57BL/6 which we use to investigate the effects on organs between a control group and a EGCG treated group at day +17. Both models have been described previously. [9, 16] Allo-BMT recipients received a combination of 25mg/kg EGCG (Enzo Life Sciences, Lörrach, Germany) and 1mg/kg quercetin (Sigma Aldrich) dissolved in DMSO (20μl total) and PBS i.p. daily or EGCG and quercetin alone (for clinical score) or DMSO only (20μl, control) respectively from day 0 to end of experiment.

### Evaluation of clinical and histopathological score

Histopathological scores were determined after the Lerner criteria. [17] Liver score was dependent on leukocyte infiltration of portal triads (grade I: <25% infiltration, grade II: <50%, grade III: <75%, grade IV: >75%). Number of animals per group: N = 10 (clinical score), N = 8 (histopathological score).

### Immunhistochemistry

For analysis of CD4 and CD8 lymphocyte infiltration of GVHD target organs, quantification of Foxp3 positive T cells in the colon and for analysis of neovascularization in liver and colon we used the protocols as described previously. [16] Number of animals: N = 8.

### Flow cytometry staining

For flow cytometry analyses at day +17 after BMT, peripheral blood, spleen and lymph nodes were harvested as described previously. [16] Single cell suspensions were stained with rat anti-mouse antibodies from BD Biosciences as follows: CD3e-APC-Cy7 (1:100), CD4-PE-Cy7 (1:800), CD8a-APC (1:200), CD25-PerCP-Cy5.5(1:200), CD11b-APC-Cy7 (1:400), B220-PerCP-Cy5.5 (1:100) or Nk1.1-PerCP-Cy5.5 (1:200). For chimerism analysis of blood and BM, antibodies against PE- Ly9.1 (1:100) and FITC-H2kb (1:50) were used. Regulatory T cell staining in blood and spleen were performed using Anti-Mouse/Rat FoxP3 Staining Set APC (eBioscience, San Diego, CA, USA) following the manufacturer´s instructions. Samples were analyzed by BD FACS Canto II (BD Biosciences) and FlowJo 7.6.5 Software (TreeStar Inc., Ashland, OR, USA).

### Glutathione and superoxide dismutase assay

For measuring glutathione in blood, colon and liver at day +17, an assay was performed with the glutathione (GSSG/GSH) detection kit (Enzo Life Sciences, East Farmingdale, NY, USA) after manufacturer´s instructions. The superoxide dismutase assay kit from cayman chemical (Ann Arbor, MI, USA) for measuring SOD activity in colon and liver was performed following the manufacturer`s instructions. Number of animals: N = 8.

### Statistics

Survival data was analyzed using the Kaplan-Meier-method and compared with the Mantel- Cox log-rank test. For statistical analysis of all other data the Student´s unpaired *t*-test was used. Values are presented as mean ± s.e.m. Values of p≤0.05 were considered statistically significant. All statistical analyses were performed using GraphPad Prism software (GraphPad Software Inc., La Jolla, CA, USA).

Additional information is available under S1 File.

Raw data are available under https://osf.io/cznuk/, PLOSONE: The green tea catechin Epigallocatechin gallate ameliorates Graft-versus-host disease.

## Results

### Plasma levels of EGCG can be elevated by quercetin and piperine

**Fig 1:**
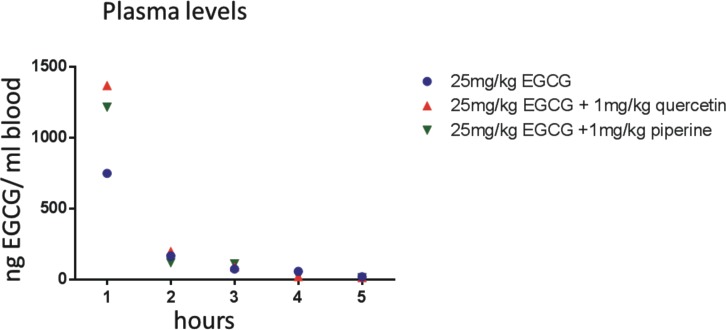
Impact of quercetin and piperine on EGCG plasma levels. Mice were i.p. injected with 25mg/kg EGCG and 1mg/kg quercetin or 1mg/kg piperine, respectively. Blood was taken after 1, 2, 3, 4 and 5 hours and analyzed by LC-MC/MC by Pharmacelsus, Saarbrücken, Germany. A typical experiment is shown.

Upon uptake, EGCG underlies a rapid chemical transformation (methylation, glucuronidation) reducing its bioavailability. [18] To prevent chemical transformation and to enhance bioavailability of EGCG, quercetin or piperine have been used. Both substances are known inhibitors of EGCG methylation. [19, 20] We were interested in the effect of quercetin and piperine on EGCG plasma levels. We used a dose of 25mg/kg EGCG, which is based on our previous experiences in animal models of colitis. [2] After injection of 25mg/kg EGCG alone or in combination with 1mg/kg quercetin or 1mg/kg piperine we assessed EGCG plasma concentrations (Fig 1). Both, the addition of quercetin or piperine increased the peak plasma levels one hour after injection (Fig 1). The mice tolerated the quercetin better than the piperine, we thererfore completed all other experiments using quercetin.

### EGCG treatment improves survival and clinical scores of allo-BMT recipients with GVHD

**Fig 2:**
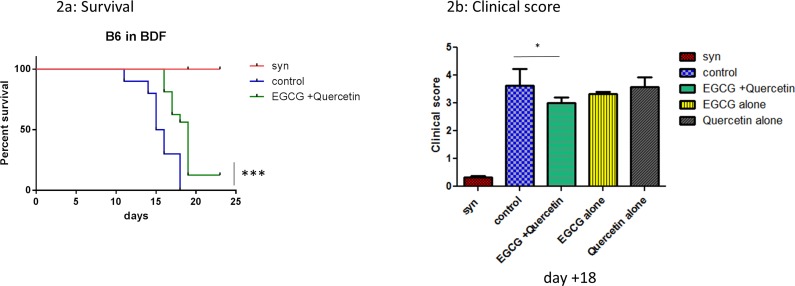
Impact of EGCG on GVHD-related mortality and clinical GVHD symptoms. (a) Impact of EGCG on survival during GVHD. 2x10^7^ BM cells and 5x10^6^ T cells from C57BL/6 (H-2K^b^) mice were injected into B6D2F1 (H-2K^d^) mice after busulfan and cyclophosphamide conditioning (4 days 20mg/kg/day busulfan, 2 days 100mg/kg/day cyclophosphamide). Significant differences were seen between EGCG treated and untreated control group (***p<0.001). (b) Impact of EGCG on clinical GVHD scores after BMT. 1,5x10^7^ BM and 2x10^6^ T cells from LP/J mice were injected into C57BL/6 mice after busulfan and cyclophosphamide conditioning (5 days 20mg/kg/day, 2 days 100mg/kg/day cyclophosphamide). EGCG +quercetin treated mice showed significantly decreased GVHD scores compared with control animals. N = 10 per group (*p<0.05).

We first studied if a prophylactic treatment with EGCG/quercetin has an impact on the course of lethal GVHD. We treated allo-BMT recipients with 25mg/kg EGCG and 1mg/kg quercetin from day 0 to the end of the experiment. We found a significantly increased survival rate in EGCG treated allo-BMT recipients vs. non-treated allo-BMT recipients during GVHD (*p*<0.001) (Fig 2A). In addition, EGCG treated allo-BMT recipients had significantly lower clinical GVHD scores as compared with the control group (p<0.05) (Fig 2B). Treatment with 25mg/kg EGCG alone resulted in slightly lower clinical scores without reaching statistical significance. When we administered 1mg/kg quercetin alone, we found no effect on GVHD (Fig 2B), demonstrating that quercetin has no major effects on GVHD in our models.

### GVHD related target organ damage is reduced in EGCG treated allo-BMT recipients

**Fig 3:**
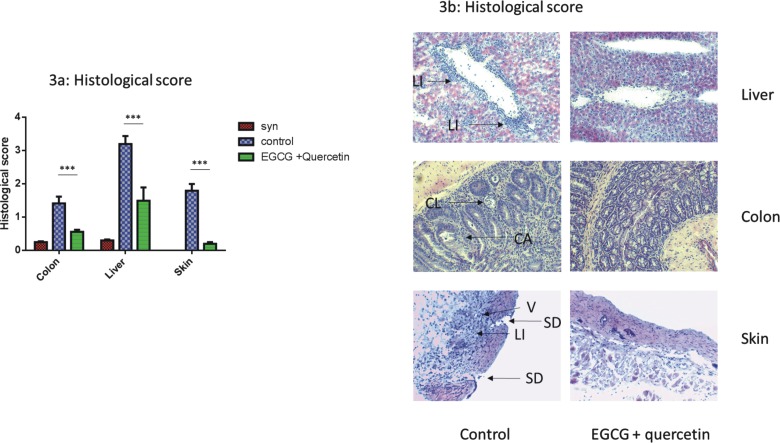
Impact of EGCG on target organ GVHD. 1,5x10^7^ BM and 2x10^6^ T cells from LP/J mice were injected into C57BL/6 mice after busulfan and cyclophosphamide conditioning. (a) Histopathological scores in GVHD target organs at day +17. H&E-stained sections of colon, liver and skin were analyzed according to Lerner [17]. Significant differences between EGCG treated and untreated control group in all target organs. Data from one representative experiment is shown, *N* = 8 per group, ***p<0.001. (b) Histopathological changes in GVHD target organs at day +17. GVHD tissue damage of untreated control group showed in the colon cryptal abscesses (CA) and crypt loss (CL), whereas EGCG treated group showed normal architecture. In the liver of the control group excessive lymphoid infiltration (LI) was observed. Liver veins of EGCG treated mice showed only minimal infiltrations around the veins. Skin of untreated control mice exhibited vacuolization of the epidermis (V), surface damage (SD) and infiltration of immune cells (LI). EGCG treated mice showed normal skin structure. Original magnification was 200x.

To analyze target organ damage we a minor mismatch GVHD model (LP→B6) [16] and applied histopathological scores after Lerner et al. [17] At day +17, we found significantly reduced histopathological GVHD scores in liver, colon and skin in EGCG treated allo-BMT recipients vs. controls without EGCG treatment (Fig 3A). In the liver, untreated control allo-BMT recipients had GVHD-associated massively increased periportal infiltration of leukocytes whereas in EGCG treated allo-BMT recipients, infiltration was only slightly increased (Fig 3B). In the colon of untreated allo-BMT recipients the crypt structure was severely disturbed with an increased number of apoptotic bodies, cryptal abscesses, crypt loss, focal necrosis and presence of inflammatory cells (Fig 3B). In contrast, EGCG treated allo-BMT recipients showed nearly normal structure with only minor changes (Fig 3B). Finally, skin histology in the EGCG treated allo-BMT recipients displayed an intact epidermal and dermal layer with normal morphology around at day +17. At the same time untreated allo-BMT recipients had severe damages including inflammatory infiltration and cysts had developed at the border of the epidermis and dermis with small infiltrates of neutrophilic granulocytes and a slight lifting of the epidermal layer.

### Immunomodulatory effects of EGCG in allo-BMT recipients during GVHD

**Fig 4:**
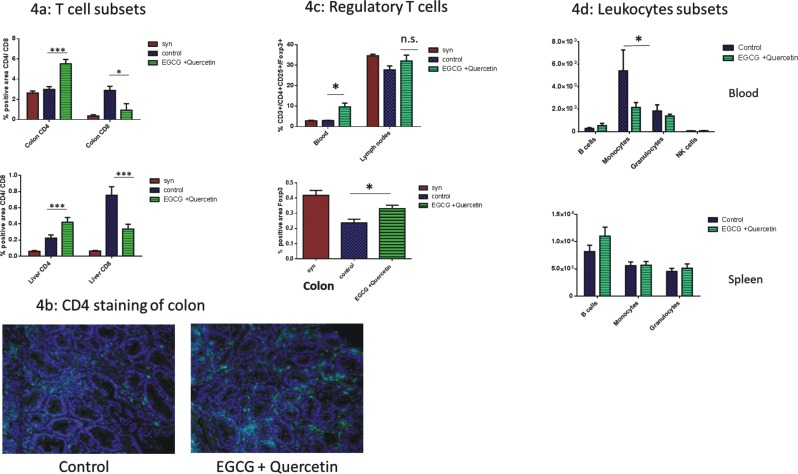
Impact of EGCG on systemic inflammation during GVHD. 1,5x10^7^ BM and 2x10^6^ T cells from LP/J mice were injected into C57BL/6 mice after busulfan and cyclophosphamide conditioning. (a) Sections of colon and liver at day +17 were stained with CD4 or CD8 antibody, and at least 6 representative pictures per animal were evaluated. CD4 positive cells were increased and CD8 positive cells were decreased as a result of EGCG treatment. (b) Representative staining of colon with anti-CD4 antibody. Abundant CD4 positive cell infiltration in colon sections of mice treated with EGCG. Sections were counterstained with 4`, 6-Diamino-2-phenylindole. (c) Regulatory T cells during GVHD. FACS analysis of blood and lymph nodes and section analysis of colon of mice treated or non-treated with EGCG. Tregs were increased in blood and colon of EGCG treated mice. (d) Leukocytes during GVHD. FACS analysis of blood and spleens of myeloid and lymphoid cells during GVHD. Within analyzed cells (B cells, monocytes, granulocytes and NK cells), monocytes were significantly decreased in the blood of EGCG treated mice. Shown is data from one representative experiment, *n* = 8 (ns = not significant, *p <0.05, **p<0.01 ***p<0.001).

Next, we were interested in possible mechanisms of EGCG-mediated inhibition of GVHD. We decided to check three known major effects of EGCG on inflammation: immunomodulation, inhibition of neovascularization and anti-oxidative effects. First we investigated immunomodulatory effects. We quantified immune cell populations at day +17 after allo-BMT in GVHD target organs using histology. We found that the CD8+ population in the colon and the liver was significantly decreased as a result of EGCG treatment (Fig 4A). Interestingly, the CD4+ population was significantly higher in target organs of the EGCG treated allo-BMT recipients vs. controls during GVHD (Fig 4A). Fig 4B shows a representative example of CD4 staining of the colon. To further characterize the CD4+ T cells we stained CD4+/FoxP3+ in colon sections. We found significantly increased CD4+/FoxP3+ staining in EGCG treated allo-BMT recipients vs. control allo-BMT recipients suggesting higher regulatory T cell (Treg) numbers (Fig 4C). Furthermore we found that numbers of CD3+/CD4+/CD25+/FoxP3+ lymphocytes (Treg) were significantly increased in the blood of the EGCG treated allo-BMT recipients (Fig 4C), but not in the lymph nodes. We conclude that EGCG modulates immune responses during GVHD leading to a higher CD4/CD8 ratio and increased regulatory T cells in the target organs as well as in blood.

Additionally we assessed the number of other immune cells found in the blood and the spleen. We found no significant differences between EGCG treated and non-treated allo-BMT recipients regarding B cells, NK cells and granulocytes. However, the number of CD11b+ monocytes was significantly decreased in the blood of EGCG treated allo-BMT recipients (Fig 4D).

### Effects of EGCG on oxidative stress during GVHD

**Fig 5:**
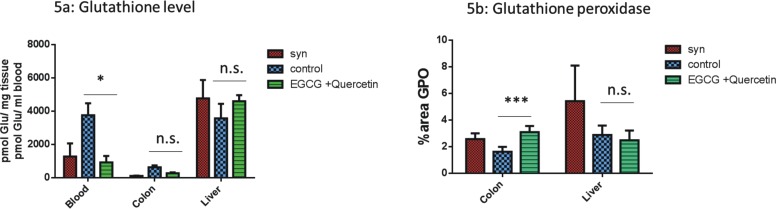
Impact of EGCG on oxidative stress during GVHD. (a) Glutathione levels during GVHD. Glutahione (GSH) was measured in blood, colon and the liver of EGCG treated and untreated allo-BMT recipients at day +17. A significantly decreased amount of glutathione in the blood was observed after treatment with EGCG. (b) GPO amounts in the colon and liver during GVHD. Sections of colon and liver at day +17 were stained with GPO antibody, and at least 6 representative pictures per animal were evaluated. A significantly decreased amount of glutathione peroxidase in the colon but not in the liver was observed after treatment with EGCG. *n* = 8 (ns = not significant, *p <0.05, **p<0.01, ***p<0.001).

Glutathione (GSH) is one of the most important antioxidants it`s known to prevent damage to cellular components caused by reactive oxygen species (ROS). It is known that when there are increased levels of ROS, glutathione is upregulated. We measured the amount of total glutathione and oxidized GSSG (glutathione disulfide) to determine the reduced GSH (total glutathione–oxidized GSSG = reduced GSH) in the blood and in the GVHD target organs colon and liver. We found significantly lower amounts of glutathione in the blood of EGCG treated allo-BMT recipients vs. controls during GVHD (Fig 5A). However, we were unable to detect major changes in glutathione concentration in GVHD target organs between EGCG treated allo-BMT recipients vs. controls (Fig 5A). A reason for this result could be that the release of the enzymes during preparation of samples that caused the glutathione to become diminished. The activity of glutathione peroxidase (GPO) was significantly increased in the colon in the EGCG treated group, whereas we could not detect any differences in the liver (Fig 5B). In summary, we found signs of diminished oxidative stress during GVHD as a result of EGCG treatment, demonstrated by significant changes of glutathione levels in the blood as well as glutathione peroxidase in the colon.

### Impact of EGCG on neovascularization during GVHD

Finally, we were interested if EGCG had effects on GVHD-related neovascularization. We harvested tissues in EGCG-treated allo-BMT recipients vs. control allo-BMT recipients at day +17. In histological sections we measured the CD31 positive area in liver and colon. We observed no significant changes in the liver (control 3,4% CD31 positive area vs. 3,28% after EGCG treatment, *p* = 0.4). In the colon (control 2,64%, EGCG group 2,89%, *p* = 0.02) changes were significant but very slight. We conclude that EGCG has moderate effects on GVHD-related neovascularization in the colon.

## Discussion

Our study provides novel evidence demonstrating that EGCG ameliorates lethal GVHD and reduces GVHD-related target organ damage. Our results are in line with previous studies demonstrating efficacy of EGCG to inhibit inflammation *in vivo*. [2, 7, 8, 12, 21] The use of EGCG in the setting of allo-HSCT is particularly interesting because of its known anti-tumor activity. EGCG is effective against a broad spectrum of tumor entities (reviewed in [22]) like colon cancer, [23] lung cancer, [24] and endometrial adenocarcinoma. [25] Numerous pathways are involved in tumor growth are affected by EGCG including suppression of NF-κB, [26] inhibition of phosphoinositide-3-kinase (PI3K), [27] Akt and STAT3 signaling, [28] down-regulation of TLR4 signal transduction, activation of 67-kDa laminin receptor (67LR) [29] and synergistic effects with anticancer drugs and micronutrients. [30]

Our results contrast a previous study investigating the *in vivo* use of EGCG in murine GVHD models. [31] Choi et al. found only minor effects of EGCG on GVHD when 100μg absolute EGCG was given to allo-BMT recipients twice a day for 21 days. Both the survival of the mice and weight development showed no significant differences between control and EGCG treated group neither in a major C57BL/6 into Balb/c mismatch model nor in a minor C57BL/6 into C57BL/6x129 F1 model. Our approach had two main differences to that of Choi et al., possibly explaining the distinct results: 1) we co-administered 1mg/kg quercetin to enhance the bioavailability of EGCG, and 2) with 25mg/kg EGCG we applied a higher dose (approximately 500μg vs.100μg EGCG / mouse). The dose of EGCG used for murine experiments has a broad range. However, in most inflammation mouse models like colitis, arthritis or EAE doses between 10mg/kg and 100mg/kg have been used as a once daily injection. [8, 32, 33] Our results are in line with another publication where donor leukocytes were *ex vivo* treated with EGCG leading to diminished GVHD in a preclinical model. [34] While this approach suggested biological relevance of EGCG for allo-HSCT it has disadvantages: 1) The *ex vivo* approach may be more difficult to translate into the clinic, and 2) the beneficial effect of EGCG on host tissues, e.g. reduction of oxidative stress, are not included.

We next investigated the mechanisms by which EGCG mediated reduction of acute GVHD. Based on previous knowledge of EGCG effects and on the pathophysiology of inflammatory diseases we were particularly interested in three major areas: 1) immunomodulation, 2) reduction of oxidative stress, and 3) anti-angiogenic effects.

First, we studied immunomodulation and we found significant changes including elevated numbers of tissue infiltrating Tregs in EGCG treated allo-BMT recipients during GVHD. We believe that this finding may be biologically important because Tregs have previously been demonstrated to be critical factors during GVHD. A reduction of regulatory T cells (Tregs) has been described during GVHD [35, 36] and an adoptive transfer of suppression of GVHD without negative impact on tumor growth has been described in pre-clinical models. [37] Currently clinical studies on the use of Tregs or substances promoting Tregs as GVHD therapies is ongoing. Interestingly, it has been shown that EGCG is able to increase Tregs *in vitro* and *in vivo*. [38] Recently, it has been described that EGCG increased the secretion of IL-2 in Balb/c mice leading to increased amounts of Tregs in the spleen. [39] EGCG also has an influence on different transcription factors leading to modified differentiation of naïve T cells. Downregulation of STAT3 and RORγt by EGCG inhibits Th17 differentiation and the suppression of activation of mTOR and subsequently HIF-1α, a metabolic check point of Th17/Treg differentiation leads to increased Treg populations. [8, 40] Besides, a modified cytokine expression pattern, caused by EGCG, directed to enhance Treg differentiation. [7, 38] In addition, an *in vitro* study has been shown that EGCG induced DNMT inhibition facilitates Foxp3 expression in naïve CD4+ T cells resulting in higher amounts of Treg. [38]

Next, we studied anti-oxidative effects of EGCG during GVHD and found diminished oxidative stress indicated by significant changes of glutathione blood levels as well as increased levels of glutathione peroxidase in the colon. It is known that oxidative stress plays a crucial role during inflammation. [41] A correlation between oxidative status in the blood and the severity of GVHD has been previously described. [13] Glutathione is one of the most important antioxidants, it prevents damage to cells and tissues and it exists in both a reduced (GSH) and oxidized (GSSG) states. A dysregulation of GSH could contribute to the development of GVHD, [42] likewise a detected imbalance of important antioxidative enzymes. [43] EGCG is a highly effective antioxidative [44] that can eliminate reactive oxygen species (ROS). [45] Furthermore, EGCG affects the enzyme activity of the antioxidant system like catalase, superoxide dismutase and glutathione peroxidase. [45] In our study, we found that EGCG had an effect on the oxidative status as well as on the anti-oxidative enzyme system, which may have contributed to the observed beneficial effects on GVHD.

Finally, we were interested in the effect of EGCG on neovascularization after allo-HSCT. We have previously demonstrated that GVHD is associated with neovascularization and have identified the inhibition of neovascularization as a therapeutic approach after allo-HSCT. [9, 10] EGCG is known to mediate anti-angiogenic effects and in different cancer entities administration of EGCG resulted in an inhibition of neovascularization and tumor growth. [5, 46] In the present study, however, we found only marginal differences in vascular density in colon, but not liver, between EGCG treated allo-BMT recipients and controls. We conclude that anti-angiogenic effects are unlikely to be mechanistically relevant for EGCG-mediated regulation of GVHD.

In summary, we demonstrate that daily administration of 25mg/kg EGCG in combination with 1mg/kg quercetin ameliorates lethal GVHD in pre-clinical models. We found significant immunomodulatory effects, including an increase of regulatory T cells, and a reduction of oxidative stress during GVHD. Our results could provide the pre-clinical data for clinical studies investigating the use of EGCG during allo-HSCT.

## Supporting Information

S1 File Supporting Information of Material and Methods. (DOCX)

S1 Fig Additional pictures for histopathological changes in GVHD target organs at day +17 in the liver. (TIF)

S2 Fig Additional pictures for histopathological changes in GVHD target organs at day +17 in the colon. (TIF)

S3 Fig Additional pictures for histopathological changes in GVHD target organs at day +17 in the skin. (TIF)

S4 Fig Additional pictures of CD4 positive cell infiltration in the colon. Pictures are shown in grey to better demonstrate CD positive cells.
(TIF)

S1 Table GVHD Scoring. Mice were individually scoring twice a week for five clinical parameters (posture, activity, fur, skin and weight loss) on a scale from 0–2. Clinical GVHD score was assessed by summation of these parameters. Animals were sacrificed when reaching a single score of 2 or exceeding GVHD score of 6.
(JPG)
