# Supplementary material for: The Green Tea Catechin Epigallocatechin Gallate Ameliorates Graft-versus-Host Disease
Source: PLoS One. 2017 Jan 19;12(1):e0169630. doi: 10.1371/journal.pone.0169630 (PMC5245838; doi:10.1371/journal.pone.0169630)

**Material and Methods**

*Mice*

Female C57BL7/6 mice, female B6D2F1 mine and female and male LP/J mice were purchased from Charles River Laboratories (Sulzfeld,Germany). Animals were 10-12 weeks old and had at least a weight of 23 g. Mice housed in the Charité University Hospital Animal Facility under pathogen-free controlled conditions and 12hr-light/dark cycle and had access to food and water *ad libitum*. All experiments were approved by the Regional Ethics Committee for Animal Research (State Office of Health and Social Affairs Berlin).

*Plasmalevel of EGCG*

We injected C57BL/6 WT mice i.p. with 25 mg/kg EGCG alone or in combination with 1 mg/kg quercetin or 1 mg/kg piperine. We collected blood from these mice after 1, 2, 3, 4 and 5 hours and prepared plasma. The plasma levels of EGCG were measured by LC-MC/MC by Pharmacelsus, Saarbrücken, Germany.

*Conditioning, allogeneic stem cell transplantation and treatment with EGCG/ Quercetin*

We used two different mouse models. The major mismatch model C57BL/6 in B6D2F1 model with high mortality to examine survival and the minor mismatch model LP/J in C57BL/6 with lower mortality to investigate differences between controls and EGCG treated groups at day +17. Both models are described before [10, 19]. In the major mismatch model female B6D2F1 mice received six days before transplantation (day -6 to day -3) 20 mg/kg busulfan (Sigma Aldrich, St. Louis, MO, USA) intraperitoneally (i.p.) daily and four days before transplantation (day -4 to day -3) mice were administered 100mg/kg/day cyclophosphamide (Sigma Aldrich). In the minor mismatch model, C57BL/6 mice received seven days (day -7 to-3) before BMT 20 mg/kg busulfan and three days before transplantation (day -5 to day -3) 100 mg/kg cyclophosphamide. Days -2 and -1 were resting days.

Mice received a combination of 25 mg/kg EGCG (Enzo Life Sciences, Lörrach, Germany) and 1 mg/kg quercetin (Sigma Aldrich) dissolved in DMSO (20 µl total) and PBS i.p. daily or DMSO only (20 µl, control) respectively from day 0 to end of experiment.

*BMT Protocol*

For allogeneic transplantation, C57BL/6 animals received 1.5 x10^7^ bone marrow (BM) cells and 2 x10^6^ splenic T cells from LP/J donor mice by tail vein injection on d0. For syngeneic transplantation, C57BL/6 mice were injected with the same number of BM cells from C57BL/6 donors. For survival experiments, C57BL/6 mice were used as donors for transplantation in B6D2F1 mice. B6D2F1 mice received 2 x 10^7^ BM cells and 5 x 10^6^ splenic cells. Splenic T cell suspension was obtained using Pan T cell isolation Kit II for mouse (Miltenyi Biotec, Bergisch Gladbach, Germany) according to manufacturer´s instructions. T cell purity was analyzed by CD3 staining by flow cytometry analysis.

*Evaluation of clinical and histopathological score*

Mice were monitored daily. Mice were individually scored twice a week for five clinical parameters (posture, activity, fur, skin and weight loss) on a scale from 0-2 (supplementary table). Clinical GVHD score was assessed by summation of these parameters. Animals were sacrificed when exceeding a score of 6. For histologic score of GVHD target organs (colon, liver, skin) mice were sacrificed on day 17 after BMT during the acute phase of GVHD.

At day +17 after BMT colon, liver and abdominal skin of EGCG treated and untreated mice were harvested and embedded for cryosection in Tissue-tek (Sakura Finetek, Alphen aan den Rijn, Netherlands). For blinded histopathological analysis of GVHD target organs, sections were stained with hematoxylin and eosin (H&E). Histopathological score were determined after Lerner criteria [20]. Liver score was depending on leukocyte infiltration of portal triads (grade I: <25% infiltration, grade II: <50%, grade III: <75%, grade IV: >75%).

*Immunhistochemistry*

For analysis of CD4 and CD8 lymphocyte infiltration of GVHD target organs, accumulation of Foxp3 positive T cells in colon and for analysis of neovascularization in liver and colon, 7 μm-sections first were blocked 1 hour with blocking buffer (PBS/3 % BSA/5 % FCS) and stained over night at 4°C with primary rat anti-mouse antibodies against CD4 (1:500), CD8a (1:500) or CD31 (1:500) from BD Biosciences or anti-mouse Foxp3-Biotin (1:500) from eBioscience. Sections were stained for 2 hours at room temperature with secondary donkey anti-rat antibody conjugated with Alexa Fluor 488 (1:1000) or Alexa Fluor 647 (1:1000) from Life Technologies. For nuclear counterstaining, 4´,6-Diamidino-2-phenylindole (DAPI) from Sigma was used. To determine CD4+/CD8+/Foxp3+/CD31+ area, at least six sections per sample were investigated with a Motic BA410 epifluorescence microscope (Motic, Hong Kong) and area was assessed by quantification of positive area to total area with a predetermined threshold using Fiji Software (<http://fiji.sc/Fiji>).

*Flow Cytometry staining*

For flow cytometry analysis at day 17 after BMT, peripheral blood was collected from retro-orbital sinus in MiniCollect EDTA tubes and spleen and lymph nodes were harvested to obtain single cell suspensions as described before [19].

Single cell suspensions were stained for 20 min at 4°C with rat anti-mouse antibodies from BD Biosciences as follows: allophycocyanin (APC)-Cy7-conjugated antibodies against CD3e (1:100), phycoerithrin (PE)-Cy7-conjugated antibodies against CD4 (1:800), APC-conjugated antibodies against CD8a (1:200), peridinin-chlorophyll (PerCP)-Cy5.5-conjugated antibodies against CD25 (1:200), APC-Cy7-conjugated CD11b (1:400) , PerCP-Cy5.5-conjugated B220 (1:100) or PerCP-Cy5.5-conjugated Nk1.1 (1:200). After staining, samples were washed twice with PBS and collected in MACS buffer. For chimerism analysis of blood and BM, antibodies against PE-conjugated antibody against Ly9.1 (1:100), and fluorescein isothiocyanate (FITC)-conjugated antibody against H2kb (1:50) were used. Regulatory T cell staining in blood and spleen were performed using Anti-Mouse/Rat FoxP3 Staining Set APC (eBioscience, San Diego, CA, USA) after manufacturer´s instructions. The included APC-conjugated antibody against FoxP3 (1:200) was used for intracellular staining. Samples were analyzed by BD FACS Canto II (BD Biosciences) and FlowJo 7.6.5 Software (TreeStar Inc., Ashland, OR, USA).


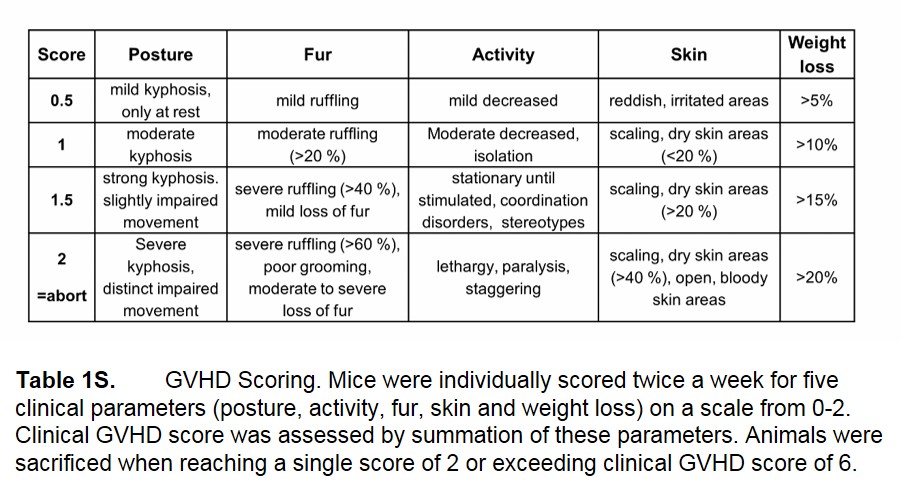

Supplement: S1 File — (DOCX) [file pone.0169630.s001.docx]
