# Supplementary figures and images for: The Green Tea Catechin Epigallocatechin Gallate Ameliorates Graft-versus-Host Disease
Source: PLoS One. 2017 Jan 19;12(1):e0169630. doi: 10.1371/journal.pone.0169630 (PMC5245838; doi:10.1371/journal.pone.0169630)

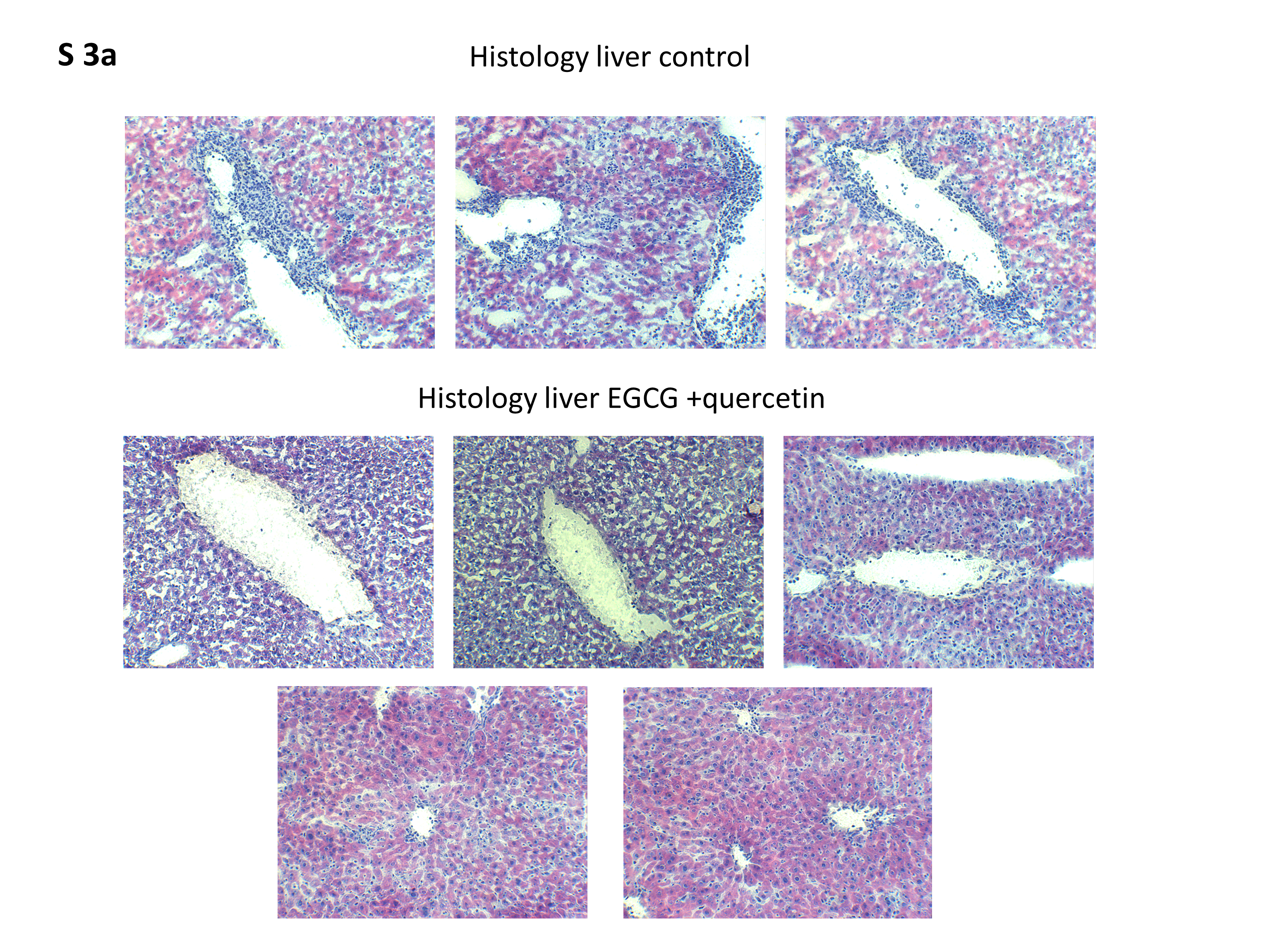

Supplement: S1 Fig — (TIF) [file pone.0169630.s002.tif]

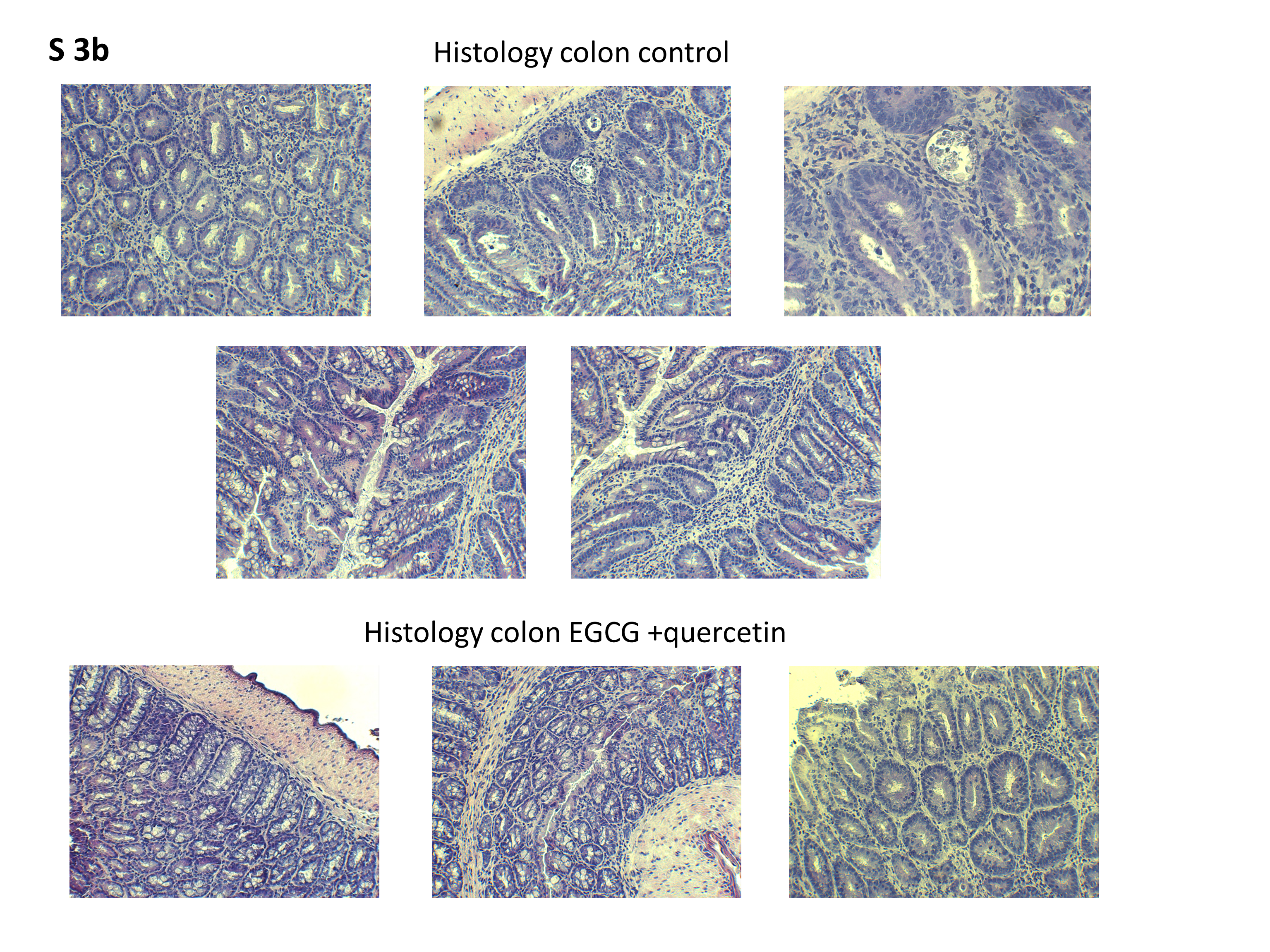

Supplement: S2 Fig — (TIF) [file pone.0169630.s003.tif]

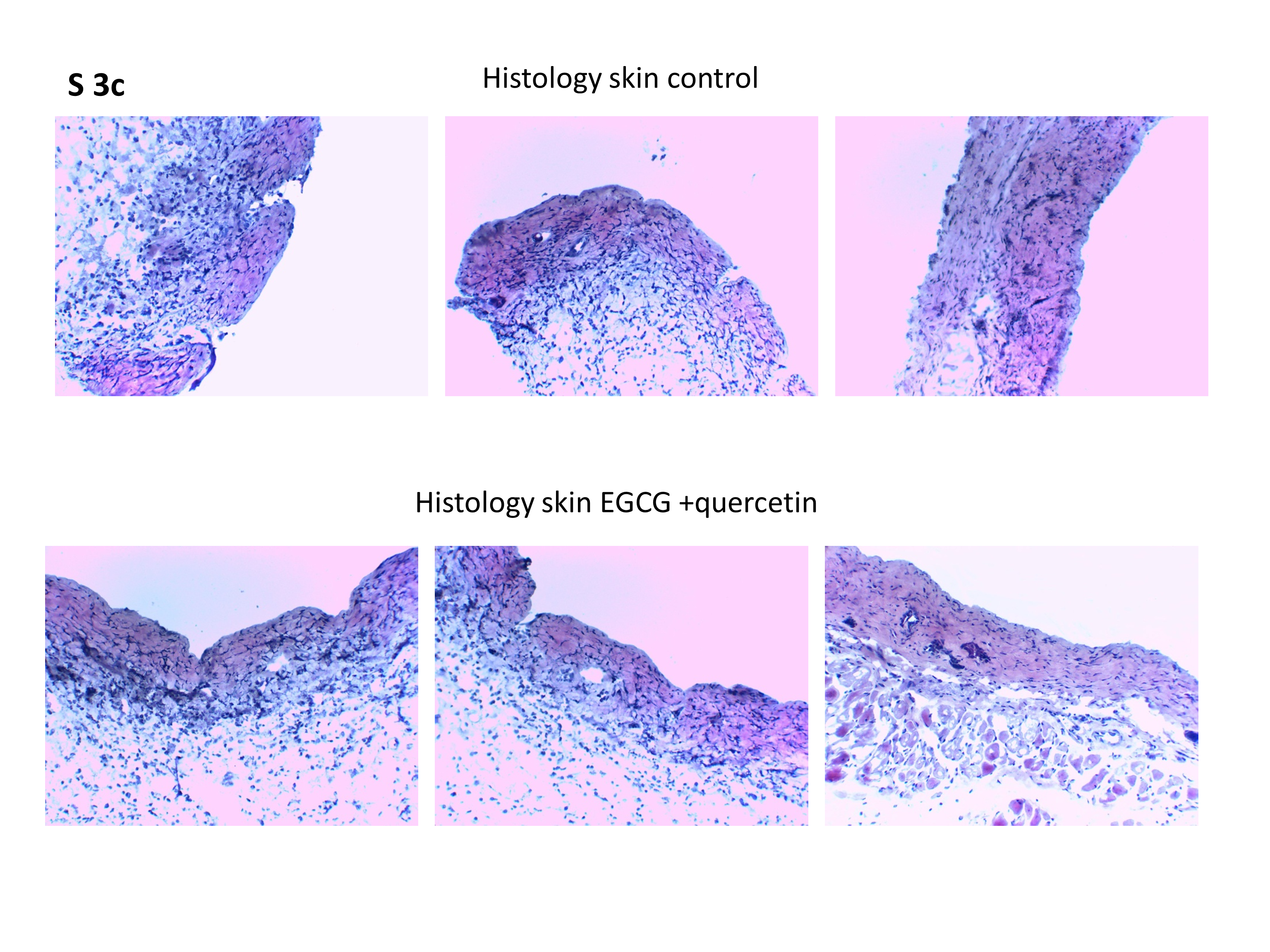

Supplement: S3 Fig — (TIF) [file pone.0169630.s004.tif]

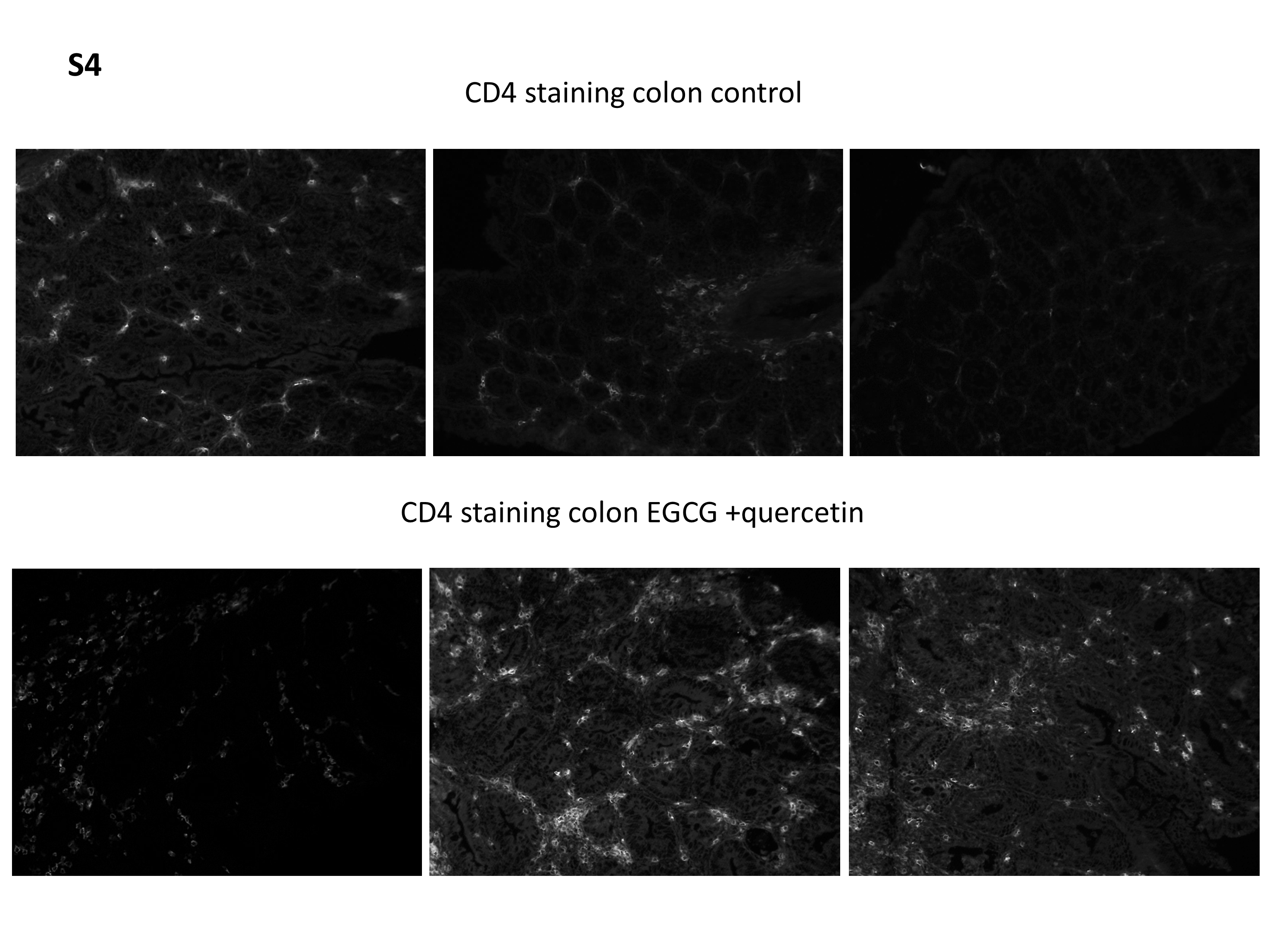

Supplement: S4 Fig — Pictures are shown in grey to better demonstrate CD positive cells. (TIF) [file pone.0169630.s005.tif]

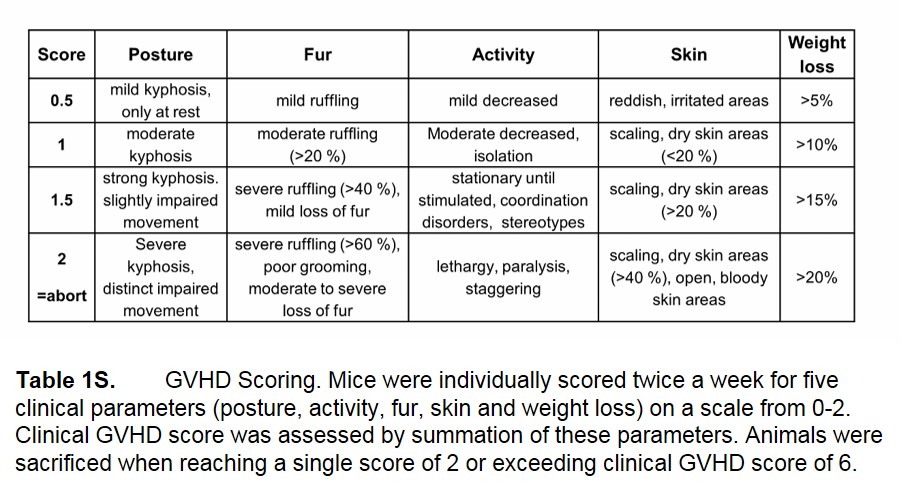

Supplement: S1 Table — Mice were individually scoring twice a week for five clinical parameters (posture, activity, fur, skin and weight loss) on a scale from 0–2. Clinical GVHD score was assessed by summation of these parameters. Animals were sacrificed when reaching a single score of 2 or exceeding GVHD score of 6. (JPG) [file pone.0169630.s006.jpg]
